# Tinnitus: well known in antiquity, highly relevant today

**DOI:** 10.1007/s00415-026-13650-2

**Published:** 2026-02-13

**Authors:** Doreen Huppert, Johannes Gerb, Filipp Filippopulos

**Affiliations:** 1 German Center for Vertigo and Balance Disorders, Klinikum Der Ludwig-Maximilians-Universität München, Marchioninistraße 15, 81377 Munich, Germany; 2 Department of Neurology, Klinikum Der Ludwig-Maximilians-Universität München, Munich, Germany

**Keywords:** Tinnitus, Menière’s disease, Vertigo, Vestibular paroxysmia, Greek antiquity, Chinese antiquity, Indian antiquity, History of neurology

## Abstract

Tinnitus, i.e., the subjective perception of sound without an external source, is a common symptom of various medical conditions. In multiple ancient medical texts, descriptions of tinnitus-like symptoms can be found, but a comprehensive overview is lacking. Here, we collect and describe sources from ancient Egypt, Mesopotamia, India, Greece, the Roman Empire, and China. Based on these text segments, we demonstrate how tinnitus has been known since antiquity. In the second part of the article, we give an overview of contemporary tinnitus care and treatment, demonstrating how a multidisciplinary diagnostic workup is crucial, and how the correct diagnosis of tinnitus is important for neurological differential diagnosis.

## Introduction

Tinnitus refers to perceived auditory sensations such as ringing, humming or buzzing, which do not have an external source. The prevalence of tinnitus is reported to be between 10 and 15% in adults, regardless of gender, and increases with age up to 70 years. In 1.5% of those affected, quality of life is significantly impaired [1]. Tinnitus is often associated with hearing loss, but it can also be a sign of central nervous disorders, tumors, or otological diseases. Given this high prevalence and the variety of underlying causes, one can postulate that tinnitus itself is not a “modern” symptom but rather existed throughout human history (similar to how, e.g., nystagmus has been known throughout antiquity, [11]). Fittingly, various ancient sources describe symptoms which would nowadays qualify as “tinnitus”. In this review, we concentrate in the first part on passages describing tinnitus in ancient Greek, Roman, Babylonian, Egyptian, Indian, and Chinese literature, providing new evidence of how this symptom was well known in ancient medicine. In view of the frequency of the symptom in modern times, we then describe the characteristics of tinnitus and its importance in common vertigo syndromes with accompanying tinnitus, such as Menière’s disease or neurovascular compression of the 8th cranial nerve. The aim of this study is to demonstrate that tinnitus is not a modern phenomenon but a known symptom across different ancient medical traditions, and to bridge these historical descriptions with contemporary clinical practice by highlighting the diagnostic and neurological relevance of tinnitus within a modern multidisciplinary framework.

## Methods

We searched original literature, translations, and existing compilations of ancient medical texts for observations and descriptions of auditory phenomena without an external source (e.g., “tinnitus”, “ringing”, “whispering”, “noise” and their linguistic derivations). Historical sources relate to cultures in Egypt, Mesopotamia, India, Greece, the Roman Empire, and China. Search terms were broad to include all potentially tinnitus-like text segments. English, French and German search terms were applied in translated literature. If available, comprehensive ancient medical texts were analyzed in the original form and employing different translations. Latin (sonitus, sensus aurium, caligo, vertigo, whirling) and Greek (ἀκούειν, ἠχος, βόμβος, ψόφος, οὖς, ἴλλιγγος) words and their derivations were used in primary sources. PubMed, Medline, and EuropePMC were used for the analysis of modern-day tinnitus care, prevalence, and importance.

## Results

### Tinnitus in antiquity

#### Greek and Roman antiquity

**Fig. 1:**
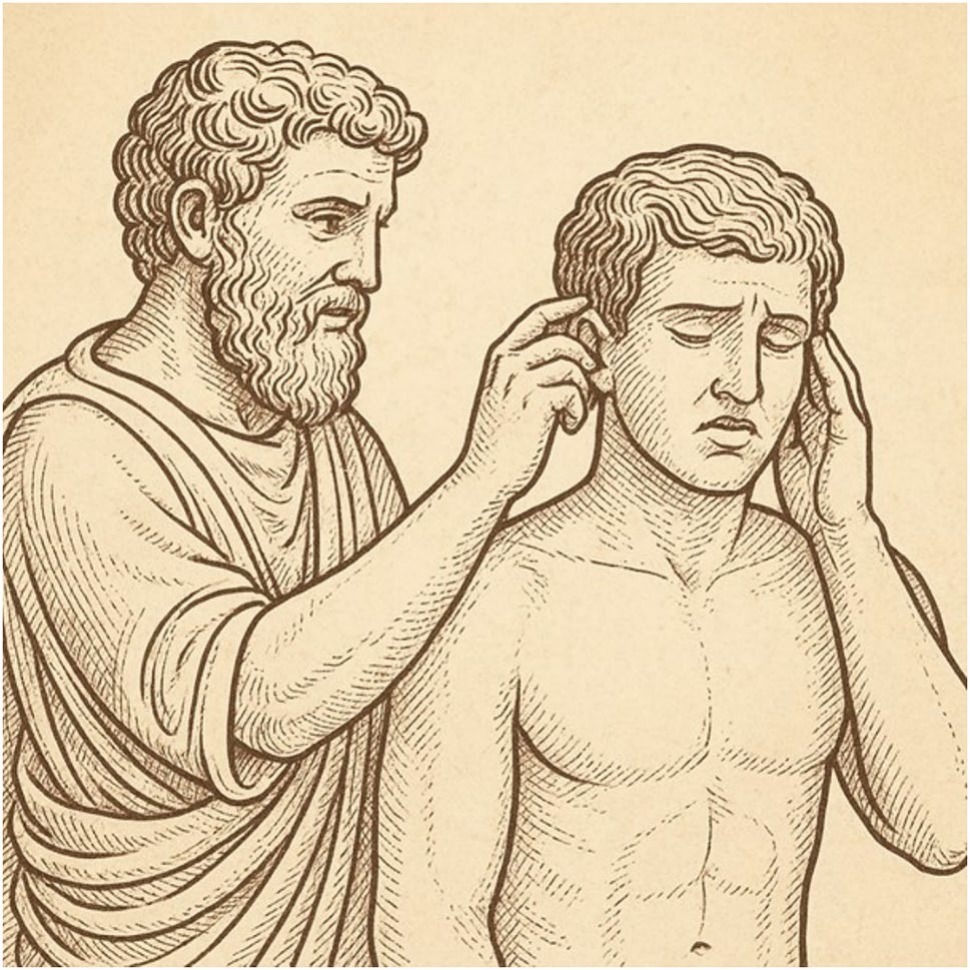
A fictional scene depicting a medical examination of a young man with tinnitus

Clinical descriptions consistent with what is today referred to as “tinnitus” was first described in ancient Greece. The term ‘tinnitus’ comes from the Latin word ‘tinnire’, which means to sound or ring. The first mention of a tinnitus-like symptom was described by ancient Greek physicians, particularly *Hippocrates* (about 460–370 BC), who discussed a variety of ear and hearing disorders in his writings. Although he did not use the term tinnitus, his descriptions of noises in the ear that impair hearing can be understood as early references to tinnitus. He reported in the ‘Corpus Hippocraticum’ on patients who heard ringing or buzzing noises in their ears and used the Greek terms ἦχος, sound, βόμβος, humming and ψόφος, quiet noise, to describe this phenomenon [17] (see Fig. 1).

In ‘Diseases 2.4’, he states: “When small blood vessels around the brain become overfilled… they begin to throb, pain spreads throughout the head, the ears begin to ring, and the patient hears nothing” (“τά ὦτα ἠχέει, ἀκούει δ’ ούδέν”) [17]. Hippocrates explains this phenomenon by a humoral imbalance, namely overfilling with blood and heat, which presses the brain against the ear, so that the air space, that is normally present there, is compromised and sounds can no longer be perceived correctly.

Further in ‘Diseases 3.1’, Hippocrates writes: “When the brain swells up… The patient’s ears are filled with ringing (“τὰ δ᾿ οὔατα ἠχῆς πλέα γίνεται”), he hears unclearly, and the vessels in his head are stretched, and throb; sometimes fevers and chills occur as well.” In this context, a “stretching of the vessels” is described to explain the ear symptoms in patients with brain swelling. Therefore, a therapeutic recommendation for “cooling agents” and to “draw off blood” is given by Hippocrates.

The Roman naturalist *Pliny the Elder* (23–79 AD) used the term ‘tinnitus’ in his work ‘Naturalis Historia’ (Sect. 23.85) to describe ringing in the ears, which is the first known use of this term: This makes him one of the first to document this condition medically, although the following passage comes from a report on celestial bodies, their movement and the sound of the spheres. Therefore, Pliny uses the expression ‘tinnitus siderum’ in a cosmological, not medical sense [9, 29].

“An sit immensus, et ideo sensum aurium excedens tantae molis rotatae vertigine adsidua sonitus, non equidem facile dixerim; non hercle magis quam circumactorum simul tinnitus siderum suosque volventium orbes, an dulcis quidam et incredibili suavitate concentus”

“Whether it is boundless and therefore surpasses the sense of hearing — the sound produced by the ceaseless whirling of such an immense mass — I would not easily dare to say; no more, by Hercules, than the simultaneous tinnitus of the stars and the orbs that revolve with them, or whether it is rather a certain harmony filled with incredible grace.”

*Aulus Cornelius Celsus* (25 BC–50 AD), a Roman encyclopedist and one of the most important medical writers of his time, describes the treatment of ear disorders in the first century AD in his work ‘De Medicina’. It is the only surviving part of the encyclopedia ‘Artes’ and is divided into eight books covering medical theory, diseases, medicines and surgery. It was written between 25 and 35 AD. Chapters 7 to 12 of Book VI deal specifically with diseases of the ear, nose and larynx [2].

“Fiunt autem et tinnitus, cum sine ulla extrinsecus causa sonitus ipse in auribus oritur.”

‘Ringing in the ears (tinnitus) also occurs when the sound arises in the ears without any external cause.’

This concise formulation is considered the classic definition of the phenomenon of tinnitus. Celsus thus describes the perception of sounds—in particular a ‘ringing’ or ‘buzzing’—independent of any external source.

*Galenos of Pergamenon* (about 129–210 AD), a Greek physician, researcher and polymath, wrote around 200 works in Greek, creating an encyclopedic body of work. He is considered one of the most important physicians of antiquity. His comprehensive teachings on the anatomy and physiology of the human body dominated the entire field of medicine until the seventeenth century. He mentions tinnitus several times, especially in his writings on ear diseases, and uses the word in its original Latin/borrowed meaning (tinnitus aurium = ringing in the ears) [22].

“Tinnitus aurium fit, cum spiritus intra canales aurium inordinate moveatur.”

“Tinnitus of the ears occurs when the pneuma moves irregularly within the passages of the ears.”

#### Chinese antiquity

The Yellow Thearch’s Classic of Internal Medicine, the *Huangdi Neijing*, is a book that lays the theoretical foundation for Chinese medicine, dating back to somewhere between the second century BC and the second century AD*.* It has two parts, the ‘Lingshu’ and the ‘Suwen’ [54] and provides lively descriptions of vertigo in combination with ringing noises (i.e., tinnitus) in different situations, e.g., at heights [3]. The book is mostly written in the form of dialogues between The Yellow Thearch, Huang Di, who is considered a cultural hero who bestowed the gift of medicine on the Chinese people, and his physicians. In the Chinese view, the flawless operation of bodily function is closely connected with all parts of the body receiving an adequate supply of specific body substances, such as blood and the vital force Qi. Various subdivisions included essences (jing), blood (xue), and Qi, conceived as implementations or various aggregate conditions of the same life force Qi. The only functional link between brain, eyes, and ears mentioned in the ‘Huangdi Neijing’ is related to hearing symptoms:

“If Qi is insufficient above, the brain is not sufficiently filled by it, the ears suffer a ringing noise, the head is bent low by it, the eyes [experience] dizziness.” [54].

Here, the brain is to be understood as the “sea of marrow”, i.e., the storage site of the marrow—a substance of the body. Thus, dizziness occurs with ear symptoms (i.e., tinnitus) and an imbalance of the head due to deficient Qi in the head. This combination of a ringing in the ears and dizziness resembles the current diagnostic criteria for MD (see below), but the pathophysiological explanations differ from our current views [14]. The pathophysiological mechanism underlying the condition was not known in ancient China, since the function of the vestibular organ had not yet been detected—the clinical history of the vestibular system only encompasses about 100 years [13].

#### Ancient Egypt, Mesopotamia, and India

In *ancient Egypt*, the ‘Ebers Papyrus’ (ca. 1550 BC, based on even older sources) contains recipes and diagnoses for ear diseases. There are passages that describe ringing in the ears or ear noises: one symptom is described as ‘a buzzing in the ear like the roar of a waterfall’.

There are instructions for treatment of the diseases with herbal oils, resins and honey, which should be inserted into the ear canal. The texts do not refer to the symptom as ‘tinnitus’, but the descriptions correspond very closely to what we understand today. Nevertheless, to date, there is no documented passage in the Ebers Papyrus that clearly describes a phenomenon similar to modern tinnitus (i.e., ‘ringing/buzzing in the ear without external cause’) [8, 36].

A second, later source from about 250 AD (possibly using earlier, older material from 200 to 300 BC) mentions several treatments for “humming”—or “storming”—in the ear, mostly involving topical ointments or herbal oils applied to the external ear, or instilled into the ear canal [10, 39].

**Fig. 2:**
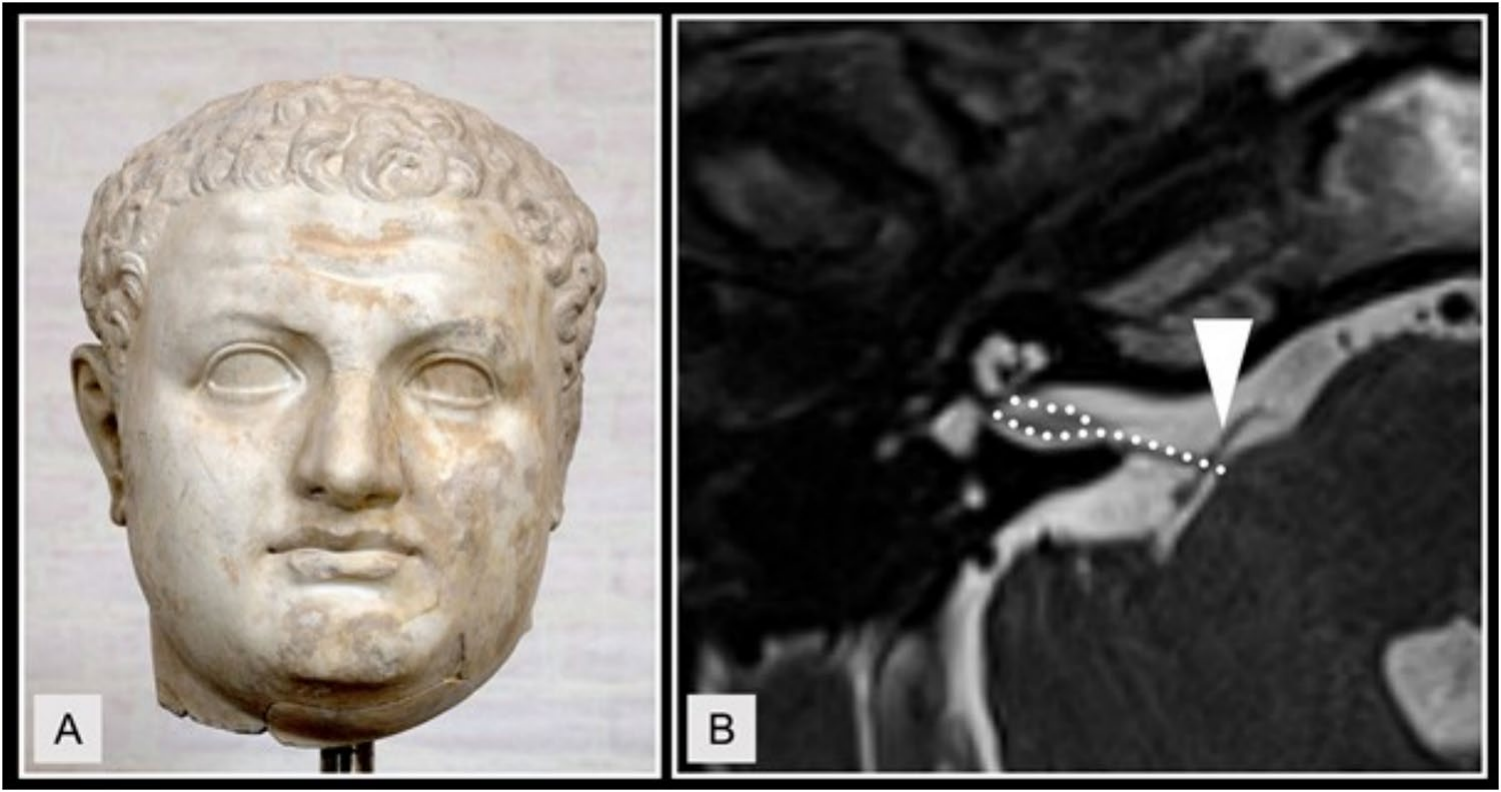
**A** Bust of the Roman Emperor Titus (39–81 AD), who, according to the Babylonian Talmud, suffered from chronic high-frequency sledgehammer-like sounds in his ear, attributed to a “gnat pecking at his brain”. **B** Axial section of T2-weighted head MRI, showing a neurovascular conflict (NVC) between the vestibulocochlear nerve (dotted lines) and arterial brainstem vasculature (arrowhead) in a patient with vestibular paroxysmia. Especially NVCs close to the root entry zone of the nerve can cause symptoms such as spells of dizziness and unilateral staccato tinnitus, often referred to as typewriter tinnitus

*Ancient Babylonian* clay tablets commonly mention “ringing”, “whispering” and “roaring” in the ear, often interpreted as a sign of demonic interference. In Babylonian medicine, a great number of different syndromes were attributed to ghosts, and despite different treatments, the affliction by the “hand of ghost” was assumed to be a common underlying mechanism in many diseases. Scurlock and Andersen note that in non-medical contexts, strange noises were also often believed to stem from ghosts; unusual noises perceived by patients, including what nowadays might be referred to as tinnitus, would therefore similarly be classified as ghost problems [41]. In the Babylonian Talmud, the following text segment on emperor Titus was considered a description of tinnitus by Dan [6] (see Fig. 2 A):

‘A gnat entered his nostril and pecked at his brain for seven years. One day Titus was passing by a blacksmith. He heard the noise of the sledgehammer and the gnat became silent. Titus thus said: “Here is the remedy”. Every day he brought a blacksmith to bang in his presence. [...] For thirty days this worked fine but then the gnat became accustomed [to the banging] and it resumed pecking.’

In Indian antiquity, there are clear descriptions of tinnitus. The physicians Charaka Samhita (approx. 400–200 BC) and Sushruta Samhita (approx. 600–200 BC) describe a pathological condition called “Karna Nada” (literally: “sound in the ear”) in major texts of Ayurveda [44] [43]. “Karna Nada” is defined by Sushruta Samhita as the perception of “ringing, buzzing, roaring, or other non-existent sounds in the ear”. Causes are linked to “dosha imbalance”, i.e., “Vata dosha”, which is associated with movement, dryness, and nervous system function [44]:

कर्णयोर्नादमाकाशं नित्यं शृणोति यो नरः ।

स विज्ञेयः कर्णनादः शुष्कवातसमुद्भवः ॥

“When a person constantly hears sounds in the ears as if from the sky, this is recognized as Karna Nada (tinnitus), arising from the dryness of Vata.”

Therapies include ‘Karna Purana’ (warm oils in the ear), ‘Nasya’ (nasal application of oils), and different dietary regulations. Charaka Samhita differentiates ‘Karna Nada’ (tinnitus) from ‘Karna Badhirya’ (deafness), clearly distinguishing these as two distinct medical conditions/entities [43]. Furthermore, the term Bhrama (giddiness, vertigo, sense of spinning) is often described together with Karna Nada (sound in the ear, i.e., tinnitus) and Karna Badhirya (hearing loss), together describing the main symptoms known today in Menière’s disease [43]:

भ्रमेण सह काननादः श्रोत्रनिरोधः च भवति।

वातसम्भवः स रोगो नाडीशुद्धौ चिकित्स्यते॥

“When vertigo (Bhrama) is accompanied by noise in the ears (Karna Nada) and hearing loss (Karna Badhirya), the disorder arises from Vata imbalance and should be treated by purifying the channels (Nadi Shuddhi).”

### Modern day tinnitus care and treatment

In this second part of the article, we focus on modern medicine, where tinnitus refers to the perception of noise in the ear without an external auditory source, also known as subjective tinnitus. In the acute manifestation, the noise in the ear is considered to be reversible within 3 months, while in the chronic manifestation, it persists for at least 3 months. Acute noise in the ear is usually accompanied by hearing loss, which can be detected audiometrically even without subjective complaints. The acute manifestation of tinnitus is therefore considered equivalent to sudden hearing loss [30]. Chronic tinnitus, on the other hand, is often based on a primary pathophysiological process in the ear and central nervous system. Neuroplastic processes, which have been detected using functional magnetic resonance imaging, lead to hyperexcitability of the central auditory system and cognitive sensitization [38]. This chronicity can trigger a vicious circle with escalating psychological stress [30]. In this case, accompanying psychiatric symptoms such as anxiety disorders and depressive syndromes are frequently found. Psychiatric comorbidity significantly impairs quality of life with high psychological distress and classifies tinnitus as decompensated. In the compensated form, this comorbidity is absent, quality of life is not significantly impaired, and psychological distress is absent or mild.

## Acute and chronic tinnitus in medical conditions

Tinnitus can occur in isolation with hearing disorders, but it can also be a symptom of various medical conditions. Specifically, otogenic disorders such as presbycusis, autoimmune diseases, or vestibular syndromes like Menière’s disease, neurological disorders such as vestibular schwannoma, meningioma or neurovascular compression of the 8th cranial nerve (vestibular paroxysmia), infections (e.g., viral infections and otitis media) or temporomandibular joint disorders are known to be associated with tinnitus, although there is no obvious pathophysiological explanation for the latter [1, 24]. Furthermore, migraine, especially vestibular migraine, can present with ear symptoms such as tinnitus and fluctuating hearing loss [45]. The pathophysiology behind this observation is still unclear; however, different mechanisms such as trigemino-vascular activation and inner ear modulation through neurotransmitters (e.g., calcitonin gene-related peptide (CGRP)) have been discussed [53].

**Fig. 3:**
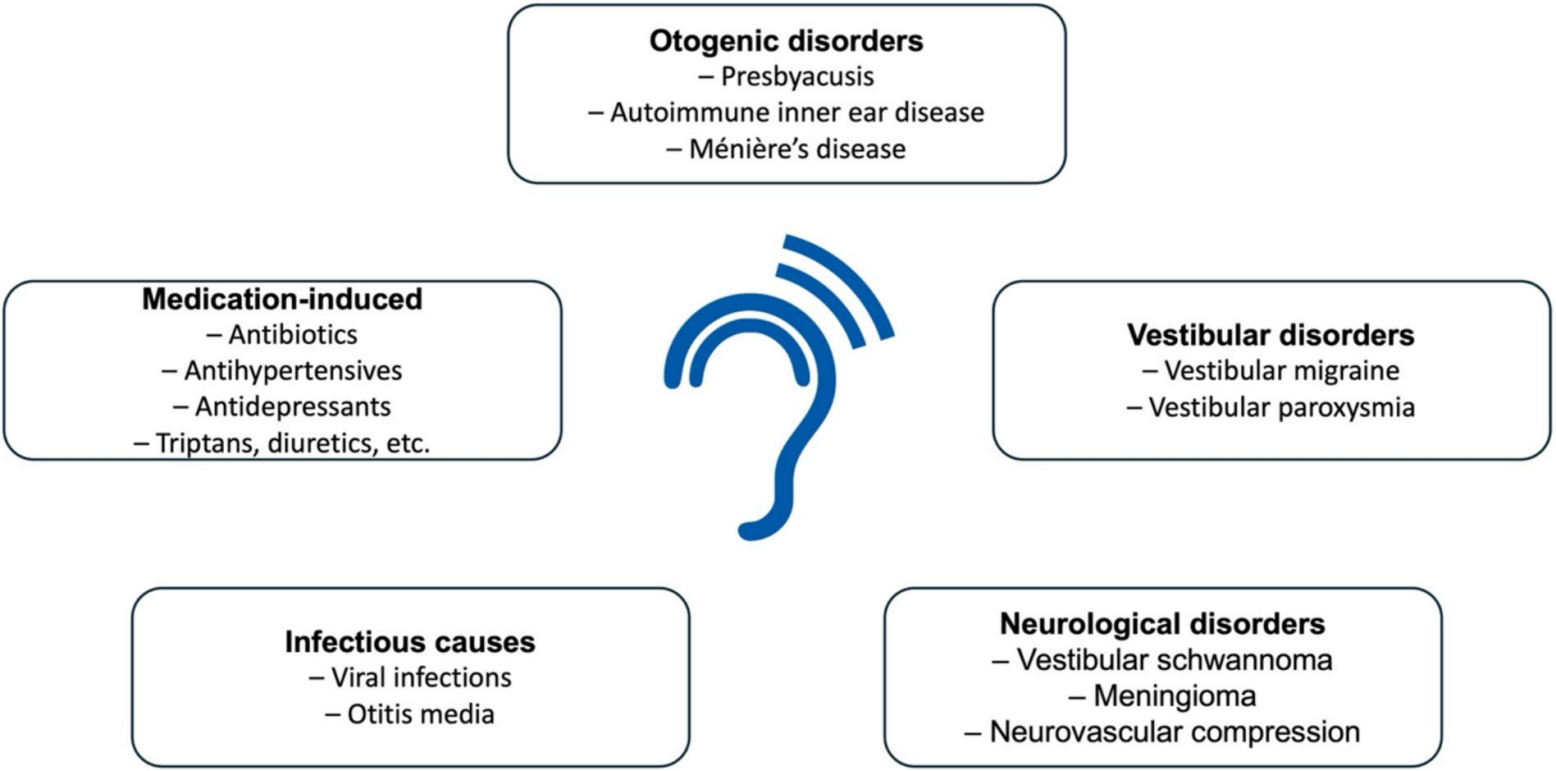
Common medical conditions associated with the occurrence of tinnitus

Lastly, some medications can trigger or exacerbate tinnitus, such as ACE inhibitors, angiotensin II antagonists, various antibiotics such as aminoglycosides and cephalosporins, antiarrhythmics, antidepressants, beta-receptor blockers, carbonic anhydrase inhibitors, chemotherapeutics, corticosteroids, immunomodulators, calcium channel blockers, loop diuretics, or triptans (see Fig. 3).

The diagnosis of acute or chronic tinnitus includes a detailed medical history, an ENT and neurological examination, tone audiometry, determination of tinnitus loudness and frequency characteristics, determination of the minimum masking level, and tympanometry. In the case of accompanying symptoms such as vertigo attacks, vestibular diagnostics or imaging (cMRT with/without contrast agent, CISS sequence, inner ear MRI) may also be necessary. Perception of clicking, rhythmic, machine-like or pulse-synchronous noises indicate that there is a source of sound within or near the ear. This clinical presentation is referred to as objective tinnitus, which requires further diagnostic workup to rule out vascular changes or neurovascular compression (see Table 1) [27, 38, 51].

**Table 1:** Diagnostic measures in case of pulse-synchronous/clicking/rhythmic tinnitus

Clinical presentation	Possible causes	Diagnostic measures
Pulse-synchronous tinnitus	Intracranial stenoses	Extra- and intracranial Doppler sonography
	Elevated jugular vein bulb	
	Dural AV fistulas	cCT or cMR angiography
	Arteriovenous malformations	(In cases of suspected vascular anomalies, also using techniques such as 4-dimensional imaging or SWI-MR imaging)
+ Auscultatory sound on the skullcap		Selective catheter angiography using digital subtraction technology
Clicking/rhythmic/staccato or machine-like tinnitus	Neuro-vascular compression of the eighth cranial nerve	cMRT including CISS or FIESTA sequences, inner ear MRT with contrast medium if necessary

The therapy is based on duration, psychological stress, and impact on quality of life. A distinction is made between the treatment of acute and chronic forms. In cases of acute tinnitus, glucocorticoid therapy is used for moderate to severe hearing loss [55]. For chronic tinnitus, the recommended therapeutic measures include educational counseling, cognitive behavioral therapy, hearing aids or cochlear implants for severe hearing loss or deafness [23, 24]. In addition, guideline-based treatment is recommended for psychiatric comorbidity [16].

## Tinnitus in vertigo syndromes

### Menière’s disease

Menière’s disease is a type of episodic vertigo syndrome that was already observed in Greek, Chinese and Indian Antiquity (see above). The full picture of MD was first described by Prosper Menière in 1861 [31]; it is the second most frequent cause of peripheral vestibular vertigo [35]. MD is characterized by recurrent attacks of vertigo lasting minutes to hours with unilateral ear symptoms (hearing loss, tinnitus, feeling of fullness). Approximately one third of patients report an increase in tinnitus, ear pressure, and/or hearing loss preceding or accompanying the abrupt vertigo attacks. Monosymptomatic attacks that are purely cochlear or vestibular can occur, particularly at the beginning of MD. Over time, most patients develop a progressive, persistent hypoacusis of the affected ear. In 2015, the diagnostic criteria for MD were reformulated by several Otolaryngological Societies working in collaboration with each other ([28], see Table 2). According to these current diagnostic criteria, audiometry is the most important instrumental examination. It requires evidence of low-level sensorineural hearing loss to identify the affected ear. This is defined as an increased bone conduction threshold of more than 30 dB in at least two consecutive frequencies below 2000 Hz compared to the other ear. Especially in the beginning, the hypoacusis resolves in the attack-free interval, so that identification of hearing loss in the above-mentioned frequencies requires instrumental testing of hearing during an acute episode of vertigo. In most cases, this leads to a relatively reliable diagnosis. Vestibular drop attacks (Tumarkin’s otolithic crisis) can also occur. They are characterized by sudden, recurring falls without loss of consciousness.

**Table 2:** Diagnostic criteria for Menière’s disease, adapted from Lopez-Escamez et al. [28]

**Menière’s disease**	A. Two or more spontaneous episodes of vertigo, each lasting 20 min to 12 h
	B. Audiometrically documented low- to medium-frequency sensorineural hearing loss in one ear, defining the affected ear on at least one occasion before, during or after one of the episodes of vertigo
	C. Fluctuating aural symptoms (hearing, tinnitus, or fullness) in the affected ear
	D. Not better accounted for by another vestibular diagnosis

The etiology and pathophysiology of MD remain unclear [33, 40, 42]; vascular, neuroimmunological, or inflammatory etiologies have been discussed. Evidence for a viral cause of MD is still equivocal [5]. The pathognomonic histopathological finding is an endolymphatic hydrops [32] that may develop as a result of a relatively increased production or decreased resorption of endolymph. The elevated pressure causes the endolymphatic membrane to rupture or the pressure-sensitive unselective cation channels to open [56]. As a result, the potassium concentration in the perilymph rises, causing the attack: first due to excitation and then to depolarization of the vestibulocochlear nerve fibers. Evidence of endolymphatic hydrops can be found in post-mortem temporal bone studies [32, 56] and also in vivo on high-resolution MRI after transtympanic gadolinium injection, since gadolinium primarily diffuses into the perilymphatic space [34], or after intravenous administration of a contrast agent [4, 12, 37]. Although almost all patients with confirmed Menière’s disease show endolymphatic hydrops, it can also be detected in other vertigo syndromes involving the inner ear, such as vestibular migraine with hearing disorders (in up to 20% of cases) [21, 37]. In cases of recurrent vertigo attacks, vestibular migraine is the most important differential diagnosis (diagnostic criteria in Refs. [25, 26]). To complicate matters, in some cases patients suffer from both Menière’s disease and vestibular migraine at the same time. A recently developed diagnostic algorithm was able to identify seven key variables, achieving a significance of 86% in differentiating between the two disease entities [15].

Many studies continue to be published on the treatment of Menière’s disease. However, most of the treatment studies are not placebo-controlled, which is particularly problematic given the high placebo effect of 50% in relation to episodes of vertigo [52]. For prophylactic therapy, betahistine dihydrochloride 24 mg is recommended (starting with 3 times one tablet and increasing to 3 × 4 tablets of 24 mg). Whether combination therapy with MAO inhibitors provides even better prophylaxis is currently the subject of ongoing studies [47].

### Vestibular paroxysmia

The main symptom is recurrent, usually spontaneous, short attacks of vertigo that occur relatively uniformly in individual patients. For a diagnosis to be made, a response to treatment with an adequate dose of a sodium channel blockers is required (i.e., ex juvantibus); otherwise, only a probable diagnosis of vestibular paroxysmia can be made (see Table 3; [49]).

**Table 3:** Diagnostic criteria for vestibular paroxysmia, adapted from Strupp et al. [49]

**Vestibular paroxysmia**	A. At least ten spontaneous episodes of vertigo B. Duration of less than 1 min C. Consistent symptoms in individual patients D. Improvement with therapy using an adequate dose of a sodium channel blocker E. Not better explained by another condition

There is a consensus in a number of publications that vascular nerve contact with the 8th cranial nerve per se has only very low specificity for the diagnosis of vestibular paroxysmia [7, 18, 19], and therefore, the diagnosis cannot be made radiologically; MRI is instead used to rule out other pathologies, however, in some cases, clinical and imaging findings complement each other (see Fig. 2 B).

In some patients, the attacks are also accompanied by hearing disturbances in the form of hyper- or hypoacusis and/or tinnitus in the affected ear [20]. If the attacks occur regularly with unilateral hearing symptoms, it is possible to identify the side likely to be affected, as has been demonstrated in individual cases [50]. Lacosamide can nowadays be applied for therapy. A case series showed a positive effect of this drug with good tolerability [48]. There is a positive placebo-controlled study with oxcarbazepine. However, the drop-out rate was 60% due to poor tolerability. Both drugs are given off-label. If no symptoms occur for 6 months, the daily dose should be reduced by 50 mg every 4 weeks and, if progress is good, the medication should be discontinued (for progression, see Ref. [46]).

## Conclusion

Tinnitus can be a debilitating symptom, often causing severe impairment of quality of life. In this narrative review, we could show that this symptom was well known already in antiquity even though caution must always be exercised when attributing ancient descriptions to modern diseases. While the treatment options varied, multiple ancient scholars correctly identified both central as well as peripheral causes and addressed the potential impact on patients’ quality of life. Cultural aspects need to be taken into account when interpreting ancient text segments; retrospective diagnosis is not always possible. In modern medicine, tinnitus requires a complex and multidisciplinary diagnostic workup, which should include specialized neuro-otological testing and imaging. Therapeutic options depend on the underlying cause, while symptomatic treatment includes cognitive behavioral therapy and sound-masking therapy.

## Data Availability

Not applicable.
